# Chemical reactivity from an activation strain perspective

**DOI:** 10.1039/d1cc02042k

**Published:** 2021-05-25

**Authors:** Pascal Vermeeren, Trevor A. Hamlin, F. Matthias Bickelhaupt

**Affiliations:** Department of Theoretical Chemistry, Amsterdam Institute of Molecular and Life Sciences (AIMMS), Amsterdam Center for Multiscale Modeling (ACMM), Vrije Universiteit Amsterdam De Boelelaan 1083 1081 HV Amsterdam The Netherlands t.a.hamlin@vu.nl f.m.bickelhaupt@vu.nl; Institute for Molecules and Materials (IMM), Radboud University Heyendaalseweg 135 6525 AJ Nijmegen The Netherlands

## Abstract

Chemical reactions are ubiquitous in the universe, they are at the core of life, and they are essential for industrial processes. The drive for a deep understanding of how something occurs, in this case, the mechanism of a chemical reaction and the factors controlling its reactivity, is intrinsically valuable and an innate quality of humans. The level of insight and degree of understanding afforded by computational chemistry cannot be understated. The activation strain model is one of the most powerful tools in our arsenal to obtain unparalleled insight into reactivity. The relative energy of interacting reactants is evaluated along a reaction energy profile and related to the rigidity of the reactants’ molecular structure and the strength of the stabilizing interactions between the deformed reactants: Δ*E*(ζ) = Δ*E*_strain_(ζ) + Δ*E*_int_(ζ). Owing to the connectedness between the activation strain model and Kohn–Sham molecular orbital theory, one is able to obtain a causal relationship between both the sterics and electronics of the reactants and their mutual reactivity. Only when this is accomplished one can eclipse the phenomenological explanations that are commonplace in the literature and textbooks and begin to rationally tune and optimize chemical transformations. We showcase how the activation strain model is the ideal tool to elucidate fundamental organic reactions, the activation of small molecules by metallylenes, and the cycloaddition reactivity of cyclic diene- and dipolarophiles.

## Abstract 2

The activation strain model is a universal tool in the chemist's arsenal to unravel the factors controlling reactivity and selectivity of any chemical transformation.

## Graphical abstract

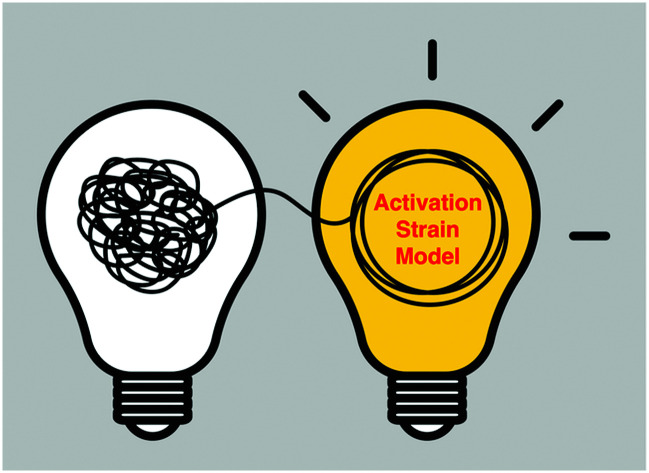

## 1. Introduction

“We must not forget that pictures and models finally have no other purpose than to serve as a framework for all the observations that are in principle possible.”— Erwin Schrödinger, Frankfurt, DE, December 1928.

Understanding the mechanism that governs a chemical reaction, allows one to rationally tune and design better reactions. This pursuit has enthralled chemists for generations and has served as a cornerstone in chemical research. One popular method to study a reaction mechanism is by exploiting quantum chemical models,^1^ which, due to the enormous advancements of computer technology in the past decades, allows one to computationally study a large variety of molecular systems and chemical processes with, for many purposes, high accuracy. To that end, many quantum chemical models have been developed to rationalize chemical reactions, such as Fukui's frontier molecular orbital (FMO) theory,^2^ valence-bond (VB) theory,^3^ and Marcus theory.^4^

Here, we present a different quantum chemical model to analyze and design chemical reactions, namely, the *activation strain model (ASM) of reactivity*, which can be used in combination with various quantum chemical software packages.^5^ The ASM aims at a deeper quantitative understanding of the physical factors that control how the reaction barrier arises in different fundamental processes. This model does so by establishing a causal relationship between the height of the reaction barrier, on one side, and the properties of the individual reactants and characteristics of the reaction mechanism. As a result, the ASM has been successfully applied by various research groups, on a wide range of chemical reactions, such as nucleophilic substitution,^6^ cycloadditions,^7^ oxidative addition,^8^ and many other processes in organic and organometallic chemistry.^9^

The ASM has been used for many years and, for that reason, has also been reviewed before.^6*a*,10^ Furthermore, we have recently written a detailed, step-by-step guide on how to perform and interpret the ASM.^11^ In this feature article, we will focus on how the ASM can be used as a tool to not only understand chemical reactivity but also to develop models to predict the outcome of chemical reactions. First, we will discuss the ASM in great detail by describing the origin of the various energy terms within this model. Thereafter, we showcase some recent advances of applying the ASM to various chemical problems, such as the competition between S_N_2 and E2 reaction,^12^ how metallylenes activate small molecules,^13^ and the cycloaddition reactivity of cycloalkenes, cycloalkynes, and cycloallenes.^14^

The activation strain model (ASM), also known as the distortion/interaction model,^15^ is a fragment-based approach to understand chemical reactivity in terms of the properties of the original reactants (*e.g.*, sterics, rigidity, bonding capability) and the characteristics of reaction mechanisms (*e.g.*, the extent of distortion reactants must undergo). In this model, the potential energy surface Δ*E*(ζ), and thus also the reaction barrier, can be decomposed into the strain energy Δ*E*_strain_(ζ) of, and the interaction energy Δ*E*_int_(ζ) between, the reactants (eqn (1)).

1 Δ*E*(ζ) = Δ*E*_strain_(ζ) + Δ*E*_int_(ζ)

The strain energy Δ*E*_strain_(ζ) is the penalty that needs to be paid in order to deform individual reactants from their equilibrium structure into the geometry they obtain at position ζ on the potential energy surface and hence directly related to the rigidity of the reactants. In general, Δ*E*_strain_(ζ) has a positive value (*i.e.*, destabilizing) and, for that reason, it is the factor that gives rise to the occurrence of the reaction barrier. In addition, this term can be further decomposed into the strain energies of the individual reactants (eqn (2)).

2 Δ*E*_strain_(ζ) = Δ*E*_strain,fragment A_(ζ) + Δ*E*_strain,fragment B_(ζ)

The interaction energy Δ*E*_int_(ζ), on the other hand, accounts for all chemical interactions that arise when the two deformed reactants are brought together from infinity to position ζ on the potential energy surface. This energy term is, therefore, directly related to the bonding capabilities and mutual interactions between the increasingly deformed reactants along the reaction pathway. In most cases, Δ*E*_int_(ζ) is negative (*i.e.*, stabling) and hence counteracts the strain energy. Often, the interaction energy is further dissected using an energy decomposition analysis, which we will later discuss in great detail.

**Fig. 1:**
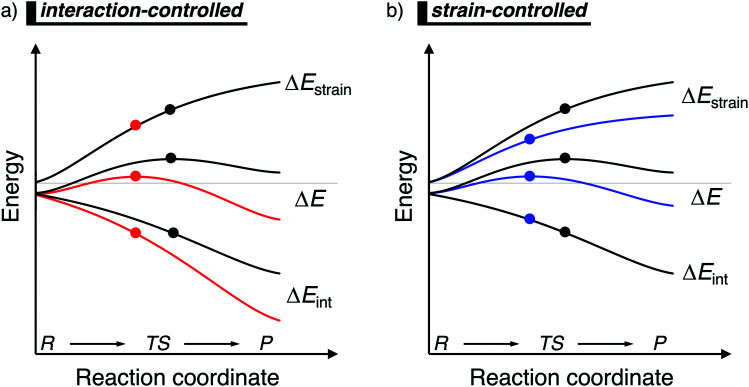
Activation strain diagram of two generic reactions (see eqn (1)). The reactivity is controlled: (a) by the interaction energy, where the red reaction has a lower barrier due to a more stabilizing interaction energy; and (b) by the strain energy, where the blue reaction has a lower barrier due to a less destabilizing strain energy (positions of TS indicated with a dot).

When the ASM is applied to a reaction profile of a chemical reaction with a central reaction barrier, which is usually obtained *via* an intrinsic reaction coordinate (IRC) calculation,^16^ all ASM energy terms start at a value close to, but not necessarily, zero (Fig. 1). A non-zero energy value is mostly observed for chemical reactions in the gas-phase, since these reactions typically start from a reactant complex, in which the fragments are already slightly distorted, *i.e.*, small Δ*E*_strain_(ζ), because of a weak interaction, *i.e.*, small Δ*E*_int_(ζ). From this point, the reactants become increasingly deformed along the reaction coordinate, leading to a continuously increasing strain energy Δ*E*_strain_(ζ). Meanwhile, the interaction between the fragments becomes stronger, leading to a consistently more stabilizing interaction energy Δ*E*_int_(ζ) along the reaction coordinate. At the position along the reaction coordinate where the destabilization of the strain terms increases with the same slope as the stabilization of the interaction energy term increases, that is, dΔ*E*_strain_(ζ)/dζ = −dΔ*E*_int_(ζ)/dζ, the derivative of the total energy Δ*E*(ζ) with respect to the reaction coordinate is zero (dΔ*E*(ζ)/dζ = 0). At this point, the reaction profile reaches either a maximum (transition state) or a minimum (reactant(complex) or product(complex)).

Besides applying the ASM along an entire reaction profile, one could also use this model to solely analyze and compare transition state structures, at which ζ = ζ^TS^ and Δ*E*^‡^ = Δ*E*^‡^_strain_ + Δ*E*^‡^_int_. This, however, should be done with great care, because the height of the reaction barrier is not only determined by the rigidity of the fragments (Δ*E*_strain_) and their mutual interactions (Δ*E*_int_), but also the slopes of these terms along the reaction coordinate. Depicted in Fig. 1a is a comparison of the activation strain analyses of two generic chemical reactions (red and black). The red reaction proceeds with a lower reaction barrier and thus the reaction is faster than for the black reaction. Performing a single-point analysis solely at the TS structures would suggest that the red reaction goes with a lower barrier due to a lower, less destabilizing strain energy because the red dot on the strain energy curve is lower than the black dot. The interaction energy at the TSs for both reactions, on the other hand, does not differ at all, because both dots are on the same vertical height. But, when one performs the analysis along the entire reaction coordinate, it becomes clear that the interaction energy of the red reaction is, at every position along the reaction coordinate, lower and hence more stabilizing than for the black reaction. In contrast, the strain energy curves of both reactions are superimposed. Thus, two alternating views of the factors controlling the reactivity emerge from the two different approaches when the TS structures of the red and black reactions occur at very different points along the reaction coordinate. Therefore, we want to emphasize that one should exhibit caution when comparing the ASM energy values of different reactions with TSs occurring at different points along the reaction coordinate.

To obtain a quantitative insight into the factors that control the interaction energy Δ*E*_int_(ζ), this term is commonly decomposed into various terms arising from different types of interactions. Such an interaction energy decomposition scheme is a powerful tool to get insight into the relative importance of the different types of contributing interactions, and for that reason, many variants have been developed which in many cases are ultimately equivalent.^3,17^ In this feature article, we use the canonical energy decomposition analysis (EDA)^18^ scheme as implemented in the Amsterdam Density Functional (ADF)^19^ package to analyze and understand the different factors controlling the interaction energy. This scheme has been selected for its transparent, easy-to-understand nature because it decomposes the interaction energy into energy components that can be understood by means of a Kohn–Sham molecular orbital (KS-MO)^20^ theory. This last point is, in our opinion, crucial, since interaction energy decomposition schemes only provide numerical data, which should not be presented as final answers to the questions but explained by quantitative methods that are directly related to causal relationships contained in and emerging from the wavefunction.

In our canonical energy decomposition analysis (EDA) scheme,^18^ the Δ*E*_int_ is defined as the sum of three individual energy terms, namely, the electrostatic interactions (Δ*V*_elstat_(ζ)), the Pauli repulsion (Δ*E*_Pauli_(ζ)), and the orbital interaction (Δ*E*_oi_(ζ)), which are all three physically meaningful and quantitatively accurate within the framework of the Kohn–Sham molecular orbital theory (eqn (3)).

3 Δ*E*_int_(ζ) = Δ*V*_elstat_(ζ) + Δ*E*_Pauli_(ζ) + Δ*E*_oi_(ζ)

Note that these energy terms should, in analogy with the ASM, be analyzed along the entire reaction coordinate, because analysis at the transition state alone might lead to skewed conclusions (*vide supra*). Next, we will discuss the individual terms contributing to the interaction energy according to our EDA scheme. In order to illustrate the origin of these terms, we consider the formation of the complex AB from two reactants, A and B, respectively. These reactants have the electronic densities ρ^A^ and ρ^B^ with corresponding wavefunctions Ψ^A^ and Ψ^B^, and total electronic fragment energies E^A^ and E^B^.

At first, the unperturbed charge distributions of reactants A and B are brought from infinity to the positions they obtain in the complex AB, resulting in the so-called promolecule which is characterized by the sum density ρ^A+B^ = ρ^A^ + ρ^B^, and the corresponding Hartree wavefunction which is a product of the unperturbed reactant wavefunctions: Ψ^H^ = Ψ^A^Ψ^B^. The associated change in energy, Δ*V*_elstat_, is simply the classical electrostatic interaction between the charge distributions of the two individual fragments:4
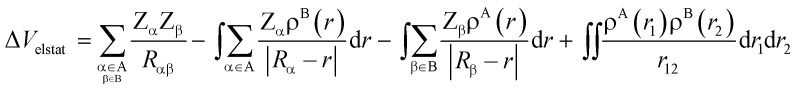


In this equation, the second and third terms describe the interaction between the attractive potential of the nuclei of one fragment with the electrons of the other, while the first and fourth terms are the repulsive nucleus–nucleus and electron–electron interactions, respectively. When the two fragments are far apart, thus when ρ^A^ and ρ^B^ do not overlap, the resulting electrostatic interaction is, in the case of neutral reactants, zero. As soon as fragments A and B start to approach each other, and ρ^A^ and ρ^B^ begin to overlap, the electrostatic interaction between the unmodified charge distributions becomes increasingly more stabilizing until the point at which the repulsion between the nuclei becomes dominant. The origin of the Δ*V*_elstat_ can be further analyzed by examining the atomic charges, such as Voronoi deformation densities,^21*a*^ Hirshfeld,^21*b*^ or multipole-derived charges,^21*c*^ and/or molecular electrostatic potential (MEP) distributions on each reactant.

In the next step, the Pauli repulsion, also known as exchange repulsion, closed-shell repulsion, or occupied–occupied orbital interaction, is calculated as the energy change of going from the Hartree wavefunction Ψ^H^, obtained in the first step, to the wavefunction Ψ^0^ = *N*Â{Ψ^H^}, which results from antisymmetrizing (operator Â) and renormalizing (constant *N*) the Hartree wavefunction and in this way correctly ensuring that Pauli's principle for fermionic wavefunctions is satisfied for the overall system. Pauli repulsion arises from electrons in either of the two fragment wavefunctions, Ψ^A^ and Ψ^B^, having the same spin and penetrating into each other's space. More specifically, it is the physical manifestation of steric effects, which are a consequence of the two-center four-electron destabilizing interactions between filled orbitals of the two reactants. For this reason, one can understand the magnitude of the Pauli repulsion by analyzing the orbital overlap between the occupied MOs on each reactant with the help of a Kohn–Sham MO analysis (Δ*E*_Pauli_ ∝ *S*^2^).^20^

In the final step of the canonical EDA, the wavefunction Ψ^0^, with corresponding electronic density ρ^0^, is allowed to relax, through occupied–virtual mixing, to the final wavefunction Ψ^AB^ and associated electronic density ρ^AB^ of the AB complex. The associated energy change constitutes the orbital interaction energy Δ*E*_oi_. This energy term is by definition stabilizing. If the two interacting reactants are closed-shell systems, the orbital interactions will consist of charge-transfer or donor–acceptor interactions between occupied orbitals on one reactant and virtual orbitals on the other (HOMO–LUMO interactions). At the same time polarization will occur, consisting of occupied–virtual mixing on one reactant due to the presence of the other. Charge transfer and polarization, however, cannot be strictly separated. The origin of the orbital interaction can be further analyzed by means of a Kohn–Sham molecular orbital analysis.^20^ The importance of an individual donor–acceptor interaction between the reactants can be ascribed to the magnitude of the orbital stabilization, which, in turn, is proportional to the HOMO–LUMO orbital overlap (actually, the interaction matrix element) squared divided by their respective orbital energy gap (Δ*E*_oi_ ∝ *S*^2^/Δε). Thus, with the help of this relationship, we can quantify and understand the importance of the individual orbital interaction mechanisms.

Furthermore, it follows from group theory that only orbitals of the same symmetry, *i.e.*, the same character under the available symmetry operations, can mix and interact. Therefore, when possible, it may be convenient to additionally decompose the orbital interaction energy, Δ*E*_oi_, into the contributions from each irreducible representation (irrep) Γ of the point group to which AB belongs, as originally introduced by Ziegler and Rauk:^22^

5 Δ*E*_oi_(ζ) = ∑_Γ_Δ*E*^Γ^_oi_(ζ)

Finally, when one supplements an exchange–correlation (XC) functional with an explicit dispersion correction Δ*E*_disp_, for instance, Grimme's dispersion correction D4,^23^ this correction term is added to Δ*E*_int_.

A potential limitation of our model is that the EDA scheme is currently not yet compatible with implicit solvation. Nevertheless, there is a workaround whereby one decomposes the solution-phase potential energy surface into the solute and solvation energies.^6*e*,*f*^ The above-described EDA can, in turn, be applied on the solute energy, which consists of the geometries as obtained by the IRC calculations with implicit solvation. Furthermore, one might perceive unimolecular reactions as a possible limitation, because this class of reactions does not consist of two clearly defined reactants. However, by judicious selection of a chemically meaningful fragmentation scheme, unimolecular reaction can, in analogy with bimolecular reactions, also be studied using this model.^9*k*,*l*^

## 2. Substitution and elimination reactions

### 2.1. Competition between S_N_2 and E2 reactions

Two archetypal reactions in organic chemistry that feature in many organic synthesis routes are the bimolecular nucleophilic substitution (S_N_2) and base-induced elimination (E2) reactions.^24^ For S_N_2 reactions, the Lewis base needs to act as a strong nucleophile, while for E2 reactions, the Lewis base is required to act as a strong protophile (*i.e.* base). A complicating factor is, however, the fact that a good nucleophile is often a strong protophile. Thus, S_N_2 reactions, which go *via* a nucleophilic attack, are always in competition with E2 reactions that go *via* a protophilic attack,^6*a*,*b*,25^ This competition opens the possibility and the necessity to actively tune the reactivity of the Lewis base toward the desired pathway in order to maximize the formation of the targeted compound and to avoid unwanted side products.

**Scheme 1:**
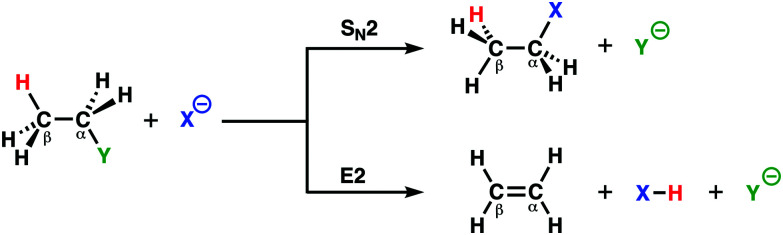
S_N_2 and E2 reaction between nucleophile X^−^ and substrate C_2_H_5_Y.

By applying the activation strain model (ASM) of reactivity, we were able to develop a general model in terms of which chemists can readily understand the dual behavior, *i.e.*, nucleophilic or protophilic, of Lewis bases in a unified manner.^12*a*^ To this end, we have analyzed the potential energy surfaces of the S_N_2 and E2 reactions of X^−^ + C_2_H_5_Y, with X, Y = F, Cl, Br, I, and At (Scheme 1), which allows us to examine the direct competition between S_N_2 and E2. Our analyses revealed the factors that determine the shape of the different potential energy surfaces from which we were able to elucidate the propensity of the Lewis base to act as a nucleophile or protophile, namely: (i) the *characteristic distortivity* of the substrate that is associated with a particular reaction pathway (S_N_2 or E2); (ii) the electron-donating capability of the Lewis base which enters into an acid–base like interaction with the substrate; and (iii) the strength of the carbon–leaving group bond. With the help of the ASM, we were able to develop the concepts of *intrinsic nucleophilicity*, *apparent nucleophilicity*, and *transition state acidity* that are associated with the different reaction pathways. These easy-to-understand concepts provide chemists with rational design principles that will enable the design of selective synthetic routes to targeted products.

Both the S_N_2 and E2 reaction barriers consistently increase, independent of the leaving group, in energy as the Lewis base X^−^ becomes less basic, along F^−^, Cl^−^, Br^−^, I^−^, and At^−^. However, the reaction barriers rise steeper along this series of Lewis bases for the E2 than for the S_N_2 reactions. This results in a switch in the preferred reaction pathway from E2, when F^−^ is the attacking Lewis base, to S_N_2 for the heavier halide anions. The reaction barrier, on the other hand, decreases for both the S_N_2 and E2 reaction pathways when the leaving group Y in the substrate C_2_H_5_Y varies along F, Cl, Br, I, and At. The reactivity trend upon changing the Lewis base shows that less basic halides, *i.e.*, those with a lower proton affinity, are both worse nucleophiles and worse protophiles, because they lead to higher barriers for the S_N_2 (nucleophilic attack) as well as the E2 (protophilic attack) reactions along the series F^−^ < Cl^−^ < Br^−^ < I^−^ < At^−^. Thus, if there were no competing E2 channels a stronger Lewis base is a better nucleophile, which is what we designate as *intrinsic nucleophilicity*. However, our analyses also show that the lowering of reaction barriers for the protophilic attack benefits more from the increased basicity of the Lewis base than that for the nucleophilic attack. This means that if the basicity becomes strong enough, the protophilic character of X^−^ always prevails. In this situation of the competition between the S_N_2 and E2 reaction channels, we speak about the *apparent nucleophilicity*. To illustrate, a weaker Lewis bases proceed with a reduced intrinsic nucleophilicity, because the reaction barriers increase, but also an enhanced apparent nucleophilicity because the S_N_2 reaction barrier is more favorable compared to the (E2 reaction barrier).

In line with its increased intrinsic nucleophilicity, stronger Lewis bases enhance, for both the S_N_2 and E2 reaction, the stabilizing interaction energy along the entire reaction coordinate, while the strain energy is minimally affected. The reason for this more stabilizing interaction energy is the stability of the X^−^ n*p* atomic orbital (AO), which decreases in energy (*i.e.*, stabilizes) along At^−^, I^−^, Br^−^, Cl^−^, and F^−^ and reduces the corresponding HOMO_Lewis base_–LUMO_substrate_ energy gap.^26^ This effect originates from the size of the AOs of the nucleophile. F^−^ has a less stable HOMO due to the compactness of fluorine AOs, which experience more destabilizing Coulomb repulsion between the electrons compared to the heavier and larger halides. A better leaving group, on the other hand, results in a weaker carbon–leaving group bond, *i.e.*, lower carbon–leaving group bond enthalpy,^27^ which manifests in less destabilizing strain energy, while the interaction energy is, in contrast, hardly affected by varying the leaving group.

**Fig. 2:**
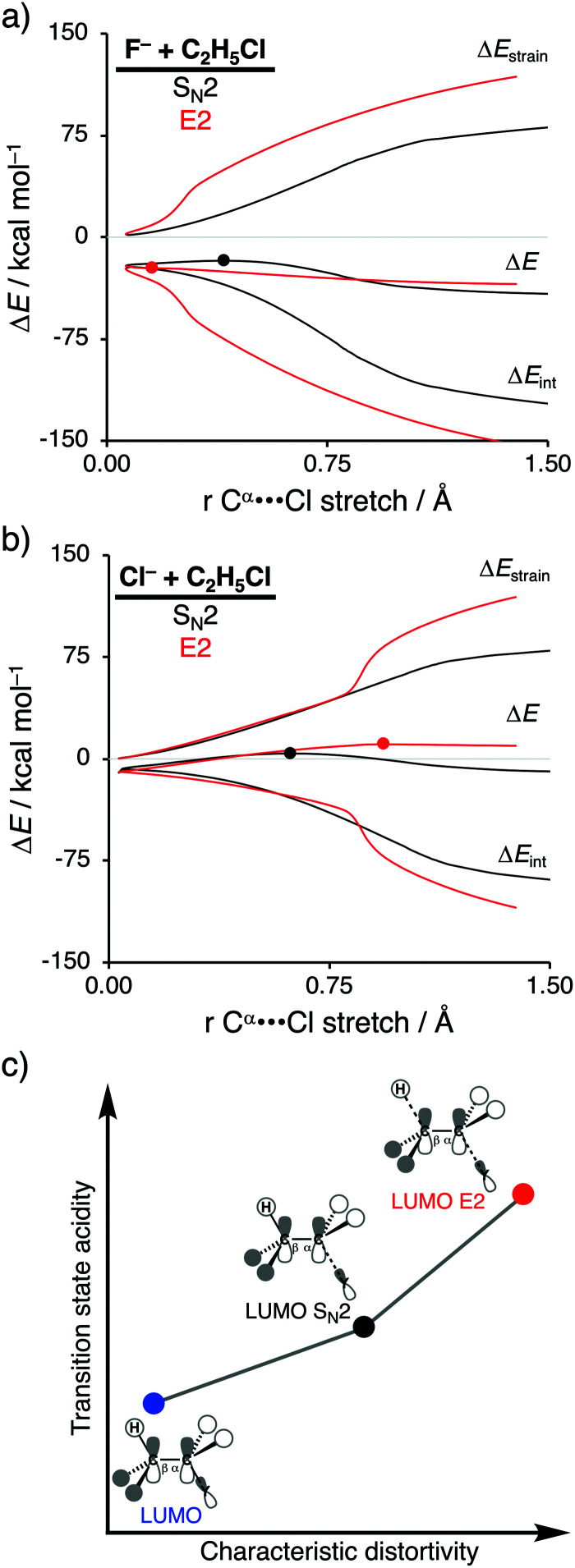
Activation strain analysis of the S_N_2 (black) and E2 (red) reaction of (a) F^−^ + C_2_H_5_Cl and (b) Cl^−^ + C_2_H_5_Cl along the reaction coordinate projected onto the C^α^⋯Cl bond stretch, computed at ZORA-OLYP/TZ2P (TS indicated with dot); (c) schematic representation of the relation between characteristic distortivity (*x*-axis) and transition state acidity (*y*-axis).

In order to directly analyze and compare the S_N_2 and E2 reaction pathways, we limit our focus to the S_N_2 and E2 pathways of the model reactions F^−^ + C_2_H_5_Cl and Cl^−^ + C_2_H_5_Cl. The former prefers to go *via* the E2 reaction pathway, while the latter prefers to follows the S_N_2 pathway. The activation strain diagrams (ASDs) show that both the strain and interaction energy curves along the E2 reaction pathway display a profound difference compared to the S_N_2 analog (Fig. 2a and b). There is, for example, a sudden jump in the strain and interaction energy curves during the E2 reaction, which can be attributed to the proton abstraction by the Lewis base that, in E2 reactions, acts as a protophile. This deprotonation of the substrate by the protophile results in a large distortion of the substrate's geometry, breaking of C–H bond, but also leads to a more stabilizing interaction (*i.e.*, X–H covalent bond formation).

As shown in Fig. 2a and b, the S_N_2 reaction pathway goes with less destabilizing strain energy than the E2 analog, because along the S_N_2 reaction pathway only one bond (C^α^–Y) is being broken, while along the E2 reaction pathway two bonds are being broken (C^α^–Y and C^β^–H). Thus, the distortion characteristic for the S_N_2 reaction pathway is by definition lower than the E2 reaction pathway. Notably, the differences in *characteristic distortivity* for both reaction pathways have an immediate effect on the electronic structure of the substrate. The LUMO of the substrate has antibonding character in both the C^α^–Y and C^β^–H bond. The characteristic distortion along the S_N_2 pathway (elongation of C^α^–Y) reduces the antibonding orbital overlap in the C^α^–Y bond, which stabilizes the LUMO of the substrate. For the E2 reaction, this stabilization of the LUMO is more significant, because the antibonding orbital overlap of both the C^α^–Y and C^β^–H bonds are being diminished making the LUMO of substrate more acidic (*i.e.*, lower-lying LUMO) than the LUMO along the S_N_2 reaction pathway. We refer to this phenomenon as the *transition state acidity* of the substrate which is stronger for E2 than S_N_2 reactions (Fig. 2c). This, ultimately, results in an intrinsically larger HOMO_Lewis base_–LUMO_substrate_ energy gap for the S_N_2 compared to the E2 pathway and, therefore, a significantly less stabilizing interaction energy between the Lewis base and the substrate, along the former reaction pathway, regardless of the Lewis base.

Changing the Lewis base from X^−^ = F^−^ to X^−^ = Cl^−^ has a profound effect on the preferred reaction pathway, shifting the above-mentioned preference from E2 for F^−^ to S_N_2 for Cl^−^. When going from F^−^ to Cl^−^ the basicity of the Lewis base is reduced, which, therefore, engages in a weaker Lewis acid–base-like interaction with the substrate for both the S_N_2 and E2 reaction pathways, reducing the intrinsic nucleophilicity. Changing the Lewis base, on the other hand, enhances the apparent nucleophilicity, because the S_N_2 barrier becomes more favorable compared to the E2 barrier. In contrast with F^−^, the less stabilizing interaction energy between Cl^−^ and the substrate is unable to overcome the characteristic distortion that occurs along the E2 reaction pathway. It is, therefore, the Lewis acid–base-like interaction between the Lewis base and the substrate that, ultimately, determines the outcome of the S_N_2 *versus* E2 competition: (i) when the Lewis base is weak (low-lying HOMO) and hence interacts weakly with the substrate, the strain determines the reactivity trend and this factor is always more favorable, *i.e.*, less destabilizing, for the pathway with the least characteristic distortion, that is, the S_N_2 reaction pathway; (ii) when the Lewis base is strong (high-lying HOMO), the more stabilizing interaction overrules the strain and determines the reactivity trend, and this factor is always more favorable, *i.e.*, more stabilizing, for the more distortive pathway with the most acidic substrate, that is, the E2 reaction pathway. These findings show that the nucleophilic or protophilic behavior of a Lewis base towards a Lewis-acidic substrate is fundamentally co-determined by the latter.

Our model can also be utilized to explain the effect of solvation on the S_N_2 *versus* E2 competition. Solvation, in general, stabilizes the lone-pair electrons of the Lewis base and, therefore, lowers the HOMO of X^−^ and reduces its electron-donating capability or, in other words, its basicity. As a result, the solvated Lewis base engages in a weaker acid–base, *i.e.*, HOMO–LUMO, interaction with the substrate and hence changes, for instance, in the case of F^−^, the preferred reaction pathway from E2 in the gas phase to S_N_2 in solution.^28^ Additionally, solvation will also enhance the apparent nucleophilicity of the weaker Lewis bases (X^−^ = Cl^−^, Br^−^, I^−^, At^−^), since it increases the E2 reaction barrier to a larger extent than the S_N_2 reaction barrier.

### 2.2. S_N_2 *versus* E2 competition of F^−^ and PH_2_^−^

The activation strain model of reactivity has shown to be a particularly useful tool to expose the underlying physical factors that control the S_N_2 *versus* E2 competition of F^−^ and PH_2_^−^ when they react with C_2_H_5_Cl (see Scheme 1, where X^−^ = F^−^ and PH_2_^−^).^12*b*^ Scott Gronert revealed that, even though it was believed that their basicities were equal, F^−^ reacts as a protophile and abstracts the β-proton of C_2_H_5_Cl *via* the E2 pathway, while PH_2_^−^ reacts as a nucleophile and attacks at the α-carbon center of C_2_H_5_Cl following the S_N_2 pathway.^29^ This opposing reactivity preference of F^−^ and PH_2_^−^ was attributed to the fact that, along the E2 pathway, proton transfer to the third-row Lewis base PH_2_^−^ involves a charge reorganization of the transferring hydrogen atom from protonic, when bonded to the substrate, to hydridic, once it is coordinated to PH_2_^−^. According to Gronert, it is this charge reorganization that increases the E2 reaction barrier, which gives rise to the observed preference for the S_N_2 pathway and, explicitly, not by the differences in thermodynamic basicity between these two Lewis bases.

The thermodynamic basicity of an anionic Lewis base in the gas-phase is usually ascribed to its proton affinity (PA), where a high proton affinity indicates a stronger Lewis base. We found that the two Lewis bases F^−^ and PH_2_^−^ do exhibit sufficiently different PAs, namely, 375.0 kcal mol^−1^ and 368.8 kcal mol^−1^, F^−^ and PH_2_^−^, respectively. This, ultimately, leads to opposite mechanistic preferences, that is, the stronger Lewis base F^−^ prefers to react *via* the E2 pathway, while the weaker Lewis base PH_2_^−^ follows the S_N_2 pathway.

**Fig. 3:**
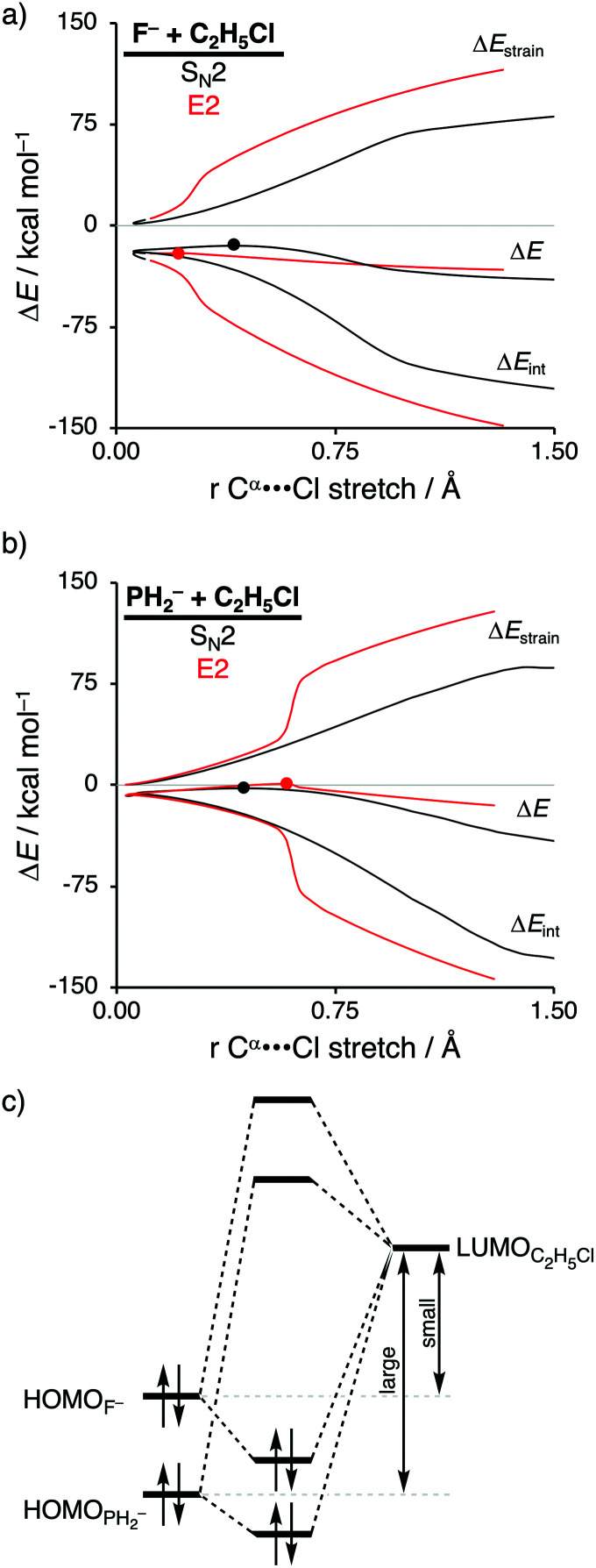
Activation strain analysis of the S_N_2 (black) and E2 (red) reaction of (a) F^−^ + C_2_H_5_Cl and (b) PH_2_^−^ + C_2_H_5_Cl along the reaction coordinate projected onto the C^α^⋯Cl bond stretch, computed at ZORA-OLYP/QZ4P (TS indicated with dot); (c) schematic molecular orbital diagram of the most important HOMO_X–_–LUMO_C2H5Cl_ orbital interaction, where X^−^ = F^−^ and PH_2_^−^.

Activation strain analyses (ASA) revealed that the preference for the E2 reaction pathway by F^−^ originates solely from a significantly more stabilizing interaction energy, which is strong enough to overcome the prior mentioned highly destabilizing characteristic distortivity associated with the E2 pathway (Fig. 3a). The mechanistic preference for the S_N_2 reaction by PH_2_^−^, on the other hand, is exclusively controlled by the strain energy, because the interaction between the weaker Lewis base PH_2_^−^ and the substrate is not able to overcome the high activation strain that is characteristic for the E2 reaction (Fig. 3b). The difference between the reactivity of these two Lewis bases can be ascribed to their differences in stability of the interacting lone pair HOMO, which, in turn, manifests into the intrinsic differences in Lewis acid–base-like interaction with the substrate (Fig. 3c). The HOMO of F^−^ is higher in energy (*i.e.*, less stable) than the corresponding HOMO of PH_2_^−^, making the former a stronger Lewis base (see also their differences in proton affinity). This difference in stability can be explained by the size of the orbitals of the Lewis base. F^−^ has a less stable HOMO due to the compactness of fluorine AOs, which experience more destabilizing Coulomb repulsion between the electrons compared to the heavier and larger HOMO_PH_2_^−^_.^26^ As a result, the more basic Lewis base F^−^ is able to engage in a stronger acid–base-like complex with the prior discussed more acidic E2 substrate (*i.e.*, small HOMO_F^−^_–LUMO_C_2_H_5_Cl_ gap), compared to the weaker base PH_2_^−^ (*i.e.*, large HOMO_PH_2_^−^_–LUMO_C_2_H_5_Cl_ gap), and is, therefore, able to generate a sufficiently stabilizing interaction with the substrate to overcome the characteristic distortion accompanied with the E2 reaction pathway. Thus, while PH_2_^−^ indeed reacts *via* a higher reaction barrier than F^−^, as found by Scott Gronert,^29^ this has little to do with the charge reorganization, but instead with the fact that PH_2_^−^ is a weaker Lewis base and hence interacts less favorable with the substrate.

### 2.3. Epoxide ring-openings *via* S_N_2 reactions under basic and acidic conditions

**Fig. 4:**
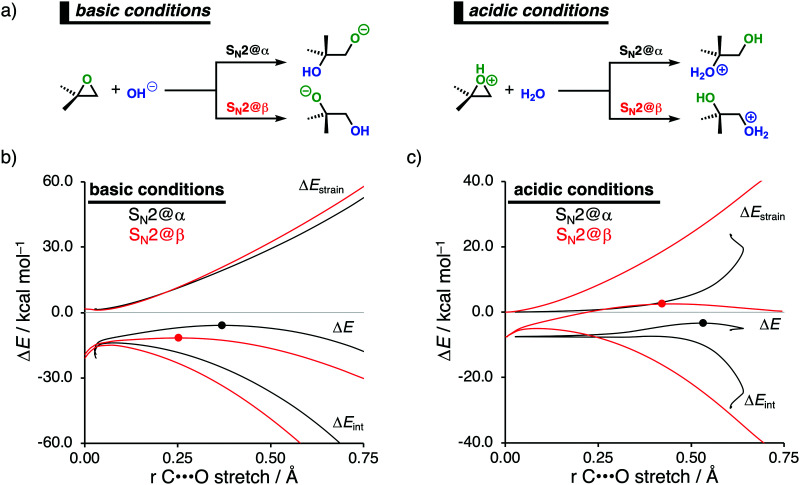
(a) Computationally analyzed epoxide ring-opening reactions under basic (left) and acidic (right) conditions. Activation strain analysis of the (b) base-catalyzed and (c) acid-catalyzed epoxide ring-opening reaction along the reaction coordinate projected onto the C⋯O bond stretch, computed at OLYP/TZ2P (TS indicated with dot).

Epoxides are an important functional group in synthetic chemistry, due to their easy availability and capability to react with a broad range of nucleophiles, which makes them valuable and versatile substrates in a wide variety of organic transformations.^30^ A well-known reaction that features epoxides is the ring-opening reaction, where the reaction conditions used during this reaction have a significant impact on the experimentally observed regioselectivity.^31^ Performing the reaction under basic conditions results in an attack of the nucleophile on the least hindered β-position of the epoxide. Under acidic conditions, on the other hand, the more substituted α-position of the epoxide will be attacked. With the help of the activation strain model, we are able to unravel the physical factors that control the regioselectivity of ring-opening reactions of the model non-symmetrical epoxide 2,2-dimethyloxirane in basic and acidic conditions (Fig. 4a).^12*c*^ To simulate basic conditions, hydroxide (OH^−^) is being used as the nucleophile, while for acidic conditions, 2,2-dimethyloxirane is protonated (2,2-dimethyloxiran-1-ium) and water (H_2_O) serves as the nucleophile.^6*b*^

Our activation strain analyses (ASA) confirm the experimentally observed regioselectivity under basic conditions, namely, the nucleophilic attack at the least sterically hindered side of the epoxide goes with the lowest reaction barrier (Fig. 4b). In line with earlier work,^31^ the regioselectivity of epoxides in base-catalyzed ring-opening reactions can be traced back to the reduced Pauli repulsion for the attack at the β-position of the epoxide. When the nucleophile attacks at the more sterically hindered α-position, the filled orbitals of the nucleophile will have a significant destabilizing occupied–occupied orbital overlap with the methyl substituents of the epoxide, which, in turn, leads to a highly destabilizing Pauli repulsion and hence less stabilizing interaction energy. Due to the absence of large substituents on the β-position of the epoxide, the nucleophile can approach this position easier and with less Pauli repulsion. This expresses itself in more stabilizing interaction energy, which is able to overrule the more destabilizing strain energy accompanying this reaction pathway. Thus, β-attack prevails in this interaction-controlled basic regime.

The regioselectivity of epoxide ring-opening reactions in acidic conditions is controlled by a completely different factor, namely, the strain energy. The nucleophilic attack in acidic conditions favors the α-position over the β-position, because the former goes with considerably less destabilizing strain energy (Fig. 4c). The weak interaction energy is, in contrast with the reaction in basic conditions, not able to overcome the regioselectivity set by the strain energy, because water is a significantly weaker nucleophile than OH^−^. The less destabilizing strain energy along the attack at the α-position is originating from the pre-distortion of the epoxide ring. In acidic conditions, the epoxide is protonated, leading to an asymmetric C–O bond elongation, because the C^α^–O bond is weaker than the C^β^–O bond. This pre-distorts the epoxide more towards the product of the attack at the α-position and translates into less strain energy along this reaction pathway. This can be seen as a manifestation of the earlier reported more stabilized carbocation-like intermediate on the α-position.^31*a*–*c*^ When the epoxide gets protonated in acidic conditions, the positive charge accumulates on the more sterically hindered α-carbon, which results in a more stabilized carbocation-like species and, as a consequence, an elongation of the epoxide's C^α^–O bond. Taken altogether, the less destabilizing strain energy overrules the less stabilizing interaction energy for the attack at the α-position compared to the β-position and the epoxide ring-opening reaction, therefore, prefers to occur at the α-position. Thus, α-attack prevails in this strain-controlled acidic regime.

## 3. Small molecule activation by metallylenes

Initiated by the seminal work of Power in 2010,^32^ the activation of small molecules by main-group elements, a field traditionally dominated by transition metal chemistry, has increased the interest of chemists. During the last decade, carbenes and their heavier Group 14 analogs (metallylenes), received growing attention, because of their similarities with transition metal catalysts.^33^ Owing to their large singlet–triplet energy gap, metallylenes have an sp^2^-hybridized lone pair orbital in the plane of the molecule and a vacant p-type orbital perpendicular to the molecular plane, which resemble the filled and empty *n*d and *n*s orbitals found in transition metal catalysts.^32^ As a result, these molecular species are able to participate in similar chemistry as their transition metal analogs, such as the activation of small molecules by oxidative insertion into the respective bond of the small molecule.^34^

**Scheme 2:**
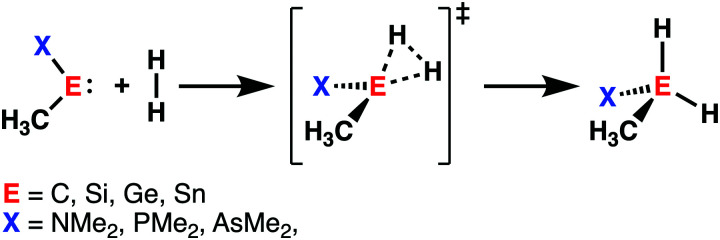
H_2_ activation by metallylenes (E = C, Si, Ge, Sn; X = NMe_2_, PMe_2_, AsMe_2_).

With the help of the activation strain model, we are able to quantify the factors that determine the trends in reactivity of the activation of dihydrogen (H_2_) by various metallylenes H_3_C–E–X, where E = C, Si, Ge, Sn, and X = NMe_2_, PMe_2_, AsMe_2_ (Scheme 2).^13^ These model metallylenes were chosen because they closely resemble experimentally accessible metallylene species.^34*a*,*c*^ We found that upon changing the central metallylene atom down in Group 14, from carbon to tin, while keeping the ligand unchanged, systematically increases the H_2_ activation barrier. Furthermore, varying the ligand X, from NMe_2_ to PMe_2_ to AsMe_2_ while keeping the central atom E constant, results in a significant lowering of the reaction barrier.

**Fig. 5:**
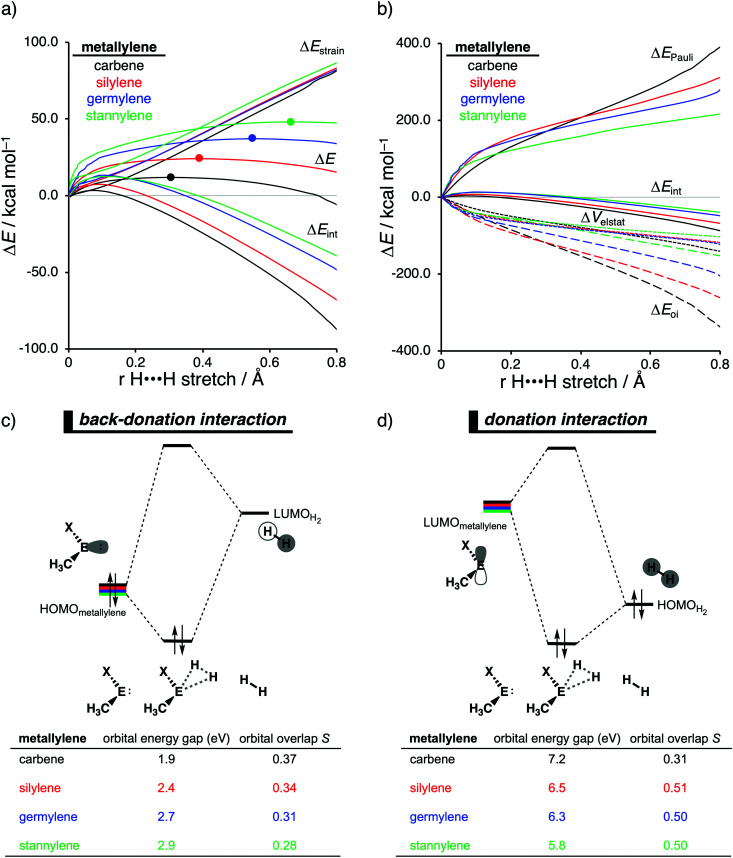
(a) Activation strain analysis and (b) energy decomposition analysis of the H_2_ bond activation by metallylenes with different Group 14 central atoms along the reaction coordinate projected onto the H⋯H bond stretch, computed at ZORA-BP86/TZ2P (TS indicated with dot). Molecular orbital diagram with the key orbital energy gap and overlap of the (c) HOMO_metallylene_–LUMO_H_2__ back-donation and (d) HOMO_H_2__–LUMO_metallylene_ donation, obtained at consistent geometries with a H⋯H bond stretch of 0.47 Å.

Our activation strain analyses revealed that the increasing reaction barrier height, on going down in Group 14 from carbon to tin while keeping the ligand X consistent, is predominantly dictated by a consistently less stabilizing interaction energy (Fig. 5a). In other words, the H_2_ activation by carbenes goes, along the entire reaction coordinate, with the most stabilizing interaction energy and hence the lowest reaction barrier. In contrast, the bond activation reactions with stannylenes engage in the least stabilizing interaction energy and, therefore, have the highest reaction barrier. The important role of the interaction energy on the observed reactivity trend prompted the analysis of the different contributors to the interaction energy using the canonical energy decomposition analysis (EDA). By performing the EDA, we established that the trend in interaction energy is almost exclusively determined by the trend in orbital interactions (Fig. 5b), which are the most stabilizing for carbenes (lowest in energy) and the least for stannylenes (highest in energy). The Pauli repulsion and electrostatic interaction, on the other hand, have a small contribution or even opposite effect on the interaction energy trend and are, therefore, not responsible for the observed reactivity trend.

The loss of stabilizing orbital interactions and hence the increasing reaction barrier, when going from carbenes to stannylenes, can be attributed to the significant weakening of the back-donation interaction between the metallylene and H_2_. Metallylenes can, in analogy with transition metal catalysts,^31^ engage *via* two orbital interaction mechanisms with H_2_, namely, the back-donation interaction, where the lone pair orbital of the metallylene (HOMO_metalyllene_) donates electrons into the σ*-orbital of H_2_ (LUMO_H2_), and the donation interaction, where the empty p-type orbital on the central atom of the metallylene (LUMO_metallylene_) accepts electrons from the σ-orbital of H_2_ (HOMO_H2_). Along the series, from carbon to tin, the HOMO_metalyllene_ becomes more stable (lower-lying HOMO) and get more diffuse, *i.e.*, increased spatial extent of the lone pair orbital on the central atom of the metallylene, which leads to a larger HOMO_metallylene_–LUMO_H2_ energy gap and a poorer orbital overlap with H_2_ (Fig. 5c). This significant weakening of the back-donation interaction is partly, but not completely, compensated by the donation interaction which becomes increasingly stronger along this series. Despite having a more favorable orbital overlap, the corresponding large LUMO_metalyllene_–HOMO_H2_ energy gap makes the donation orbital interaction mechanism not sufficiently stabilizing to overrule the trend dictated by the back-donation interaction (Fig. 5d). In summary, the strong back-donation interaction of carbenes induces a significant stabilizing orbital interaction energy, which manifests in a more favorable interaction energy and, ultimately, a lower reaction barrier. The back-donation interaction becomes, going down Group 14, less pronounced and, therefore, results in reduced orbital interactions and, as a consequence, a higher reaction barrier for the activation of H_2_.

**Fig. 6:**
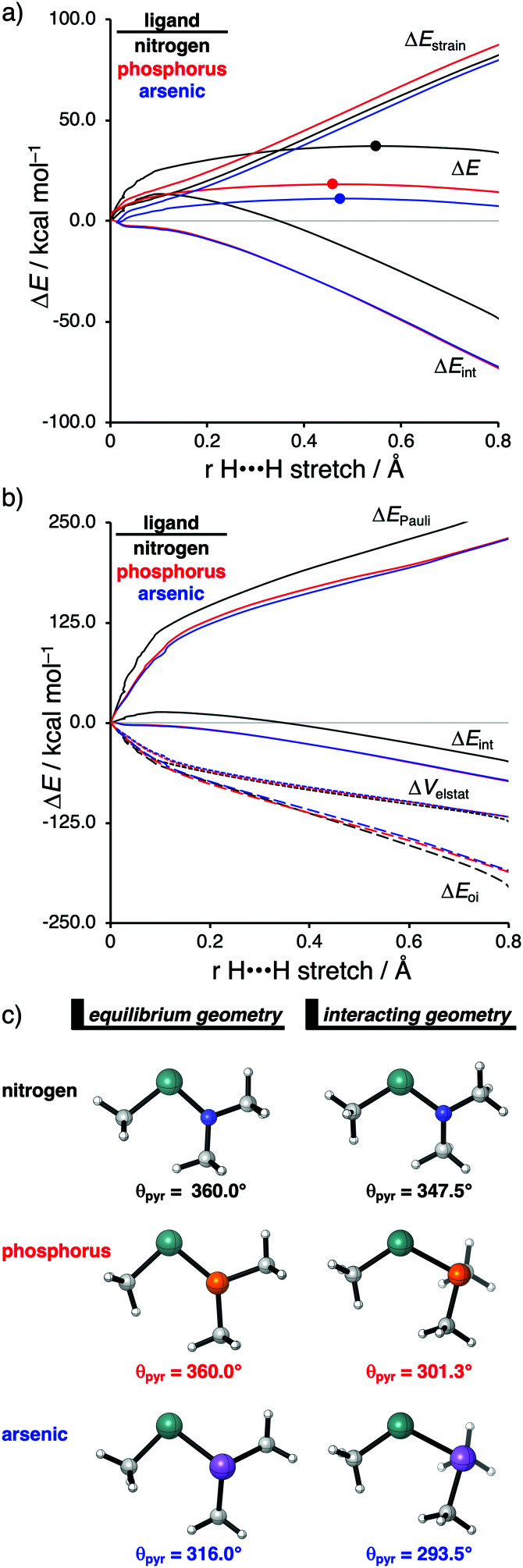
(a) Activation strain analysis and (b) energy decomposition analysis of H_2_ activation by metallylenes with different group-15 ligands along the reaction coordinate projected onto the H⋯H bond stretch, computed at ZORA-BP86/TZ2P (TS indicated with a dot). (c) Representation of the metallylene in its equilibrium and interacting geometry (pyramidalization angle θ_pyr_ = θ_C–X–E_ + θ_E–X–C_ + θ_C–X–C_).

The effect upon changing the Group 15 ligand, from nitrogen to phosphorus to arsenic, on the reactivity of the metallylene towards the activation of H_2_ is controlled by completely different physical factors, namely, the strain energy and Pauli repulsion. The activation strain diagram in Fig. 6a displays that the reaction barrier lowers when the Group 15 ligand changes from nitrogen to phosphorus to arsenic. The high reaction barrier of the nitrogen-ligated metallylene is mainly originating from the significantly less stabilizing interaction energy, which can directly be related to the highly destabilizing Pauli repulsion between the filled orbitals of the metallylene and H_2_ (Fig. 6b). The differences in reactivity between the metallylenes with a phosphorus and arsenic ligand, on the other hand, can exclusively be attributed to their difference in strain energy (interaction energy curves are superimposed), and this appears to be due to the favorable pre-distortion of the arsenic ligand (Fig. 6c). The phosphorus ligand is trigonal planar in the equilibrium geometry of the metallylene, due to the strong hyperconjugation interaction between the empty *n*p atomic orbital (AO) of the central Group 14 atom of the metallylene and the filled 3p AO of phosphorus ligand. During the course of the reaction, however, the phosphorus ligand must pyrimidalize and, therefore, diminishes the stabilizing hyperconjugation interaction. In contrast, the arsenic ligand is already pyramidal in the equilibrium geometry of the metallylene, *i.e.*, favorably pre-distorted, and, therefore, experiences less destabilizing activation strain along the reaction.

In line with the trend in strain energy, the vast difference in Pauli repulsion between the metallylenes with a nitrogen ligand, on one hand, and the metallylenes with a phosphorus and arsenic ligand, on the other, can be explained by the pyramidalization of the ligand. As mentioned above, the phosphorus and arsenic ligands pyrimidalize as the H_2_ bond activation reaction progresses. Pyramidalization of the ligand also has an effect on the electronic structure of the metallylene as it polarizes the occupied orbitals away from the central Group 14 atom of the metallylene and results in a well-defined *n*p lone pair orbital lobe on the Group 15 ligand. As a result, less orbital amplitude is pointing towards the incoming H_2_, which reduces the repulsive occupied–occupied orbital overlap between the metallylene and H_2_. The nitrogen ligand, on the other hand, undergoes a significantly smaller deformation over the course of the reaction and retains the hyperconjugation interaction, leading to a large filled-orbital lobe on the central Group 14 atom of the metallylene that engages in a larger occupied–occupied orbital overlap with H_2_. In all, the loss of hyperconjugation interaction upon pyramidalization of the Group 15 ligand results in less occupied–occupied orbital overlap and, ultimately, to a less destabilizing Pauli repulsion and a lower reaction barrier, for the reaction involving metallylenes with phosphorus and arsenic ligands compared to a metallylene with a nitrogen ligand.

## 4. Cycloaddition reactivity of cyclic diene- and dipolarophiles

**Scheme 3:**
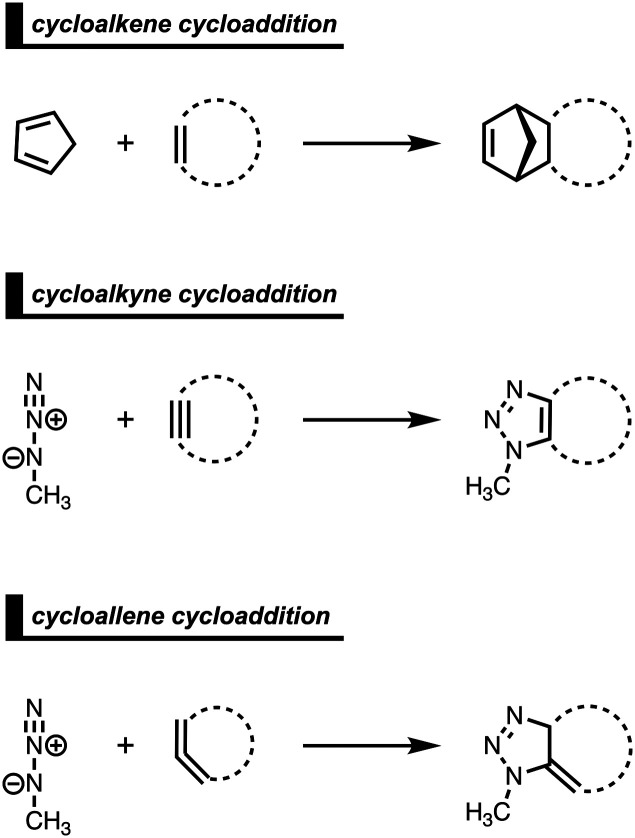
Studied cycloadditions of cycloalkenes, cycloalkynes, and cycloallenes.

Strained cyclic dienophiles and dipolarophiles (*i.e.*, cycloalkenes, cycloalkynes, and cycloallenes) are reactive partners in cycloaddition reactions (Scheme 3). The cycloaddition reactivities of cycloalkenes, cycloalkynes, and cycloallenes span several orders of magnitude and increase as the ring size decreases. The strain-promoted reactions of cycloalkenes and cycloalkynes are important in bioorthogonal chemistry for the rapid and selective *in vitro* and *in vivo* labeling of biomolecules.^35^ Historically, this reactivity enhancement for highly strained cyclic diene- and dipolarophiles has been attributed to their pre-distorted geometry that requires less distortion to reach the transition state geometry.^36^ In the following three cases, we show how strained cycloalkenes,^14*a*^ cycloalkynes,^14*b*^ and cycloallenes^14*c*^ go with accelerated cycloaddition and we reveal which physical mechanism is really behind this enhanced reactivity.

### 4.1. Cycloaddition reactivity of cycloalkenes

**Fig. 7:**
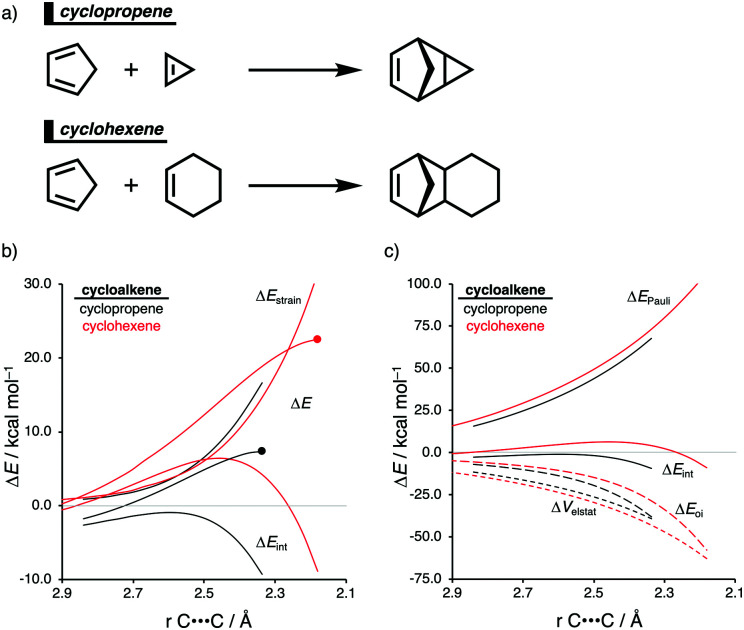
(a) Computationally analyzed Diels–Alder reactions between cyclopentadiene and cyclopropene (top) and cyclohexene (bottom). (b) Activation strain and (c) energy decomposition analyses of the cycloaddition reactions of cyclopentadiene with cyclopropene and cyclohexene, where the energy values are projected onto the average distance of the newly forming C⋯C bonds computed at M06-2X/TZ2P//M06-2X/6-31+G(d) (TS indicated with dot).

The Diels–Alder (DA) reaction between cyclopentadiene and cyclopropene is 11 orders of magnitude faster than the analogous reaction where cyclohexene acts as the dienophile. Using the activation strain model, we were able to quantify the physical factors leading to this immense difference in reactivity.^14*a*^ It is evident that the reactivity differences between cyclopropane and cyclohexene with cyclopentadiene originate from the significant differences in the interaction energy between reactants along the reaction coordinate (Fig. 7b). The strain energy, on the other hand, is nearly identical for the DA reaction involving cyclopropene and the analogous reaction with cyclohexene. The different contributors to the interaction energy were further analyzed by means of the canonical energy decomposition analysis and revealed that it is the orbital interactions that dominate the differences in the interaction energies (Fig. 7c). The Pauli repulsion and electrostatic interaction, in contrast, have an opposite effect or only a small contribution to the interaction energy trend and are, for that reason, not responsible for the observed reactivity trend.

**Fig. 8:**
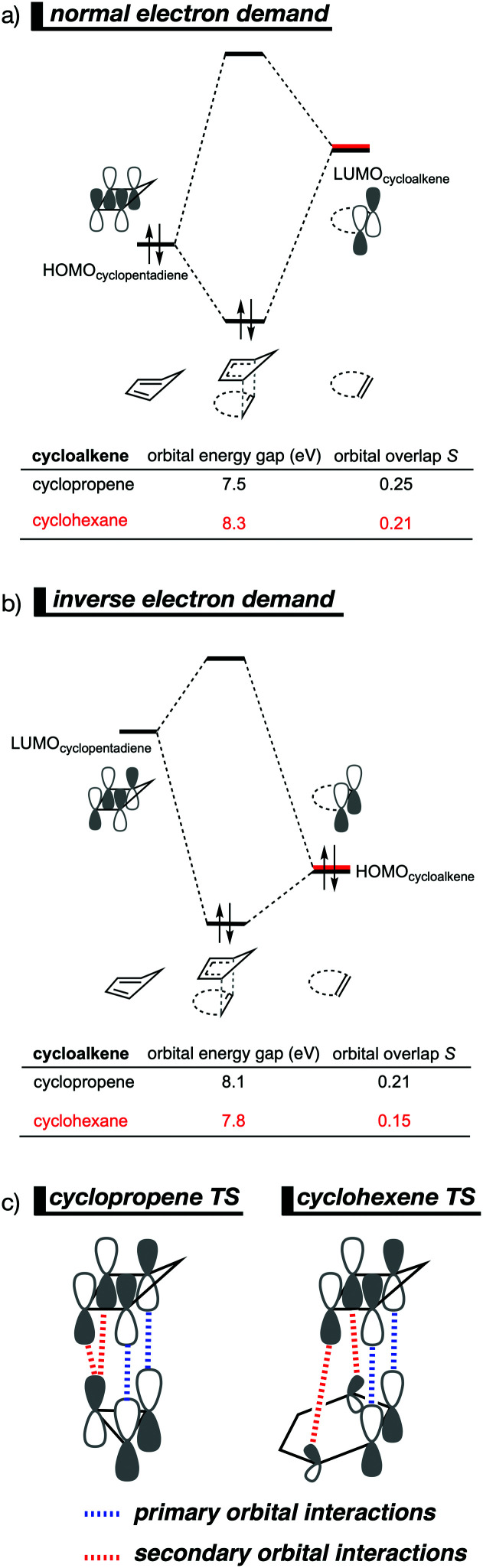
Molecular orbital diagram with orbital energies and overlap for (a) normal electron demand (NED) HOMO_cyclopentadiene_–LUMO_cycloalkene_ interaction; and for the (b) inverse electron demand (IED) LUMO_cyclopentadiene_–HOMO_cycloalkene_ interaction of the cycloaddition reactions of cyclopentadiene with cyclopropene and cyclohexene, computed on consistent geometries with an average newly forming C⋯C bonds bond distance of 2.32 Å at M06-2X/TZ2P//M06-2X/6-31+G(d). (c) Schematic representation of the secondary orbital interactions (SOI) between cyclopentadiene and the cycloalkene.

By utilizing the Kohn–Sham molecular orbital analyses, we were able to quantify the origin of the differences in orbital interactions that emerges from our quantitative energy decomposition analysis, which, ultimately, determine the reactivity trend. We found that both the normal electron demand (NED), HOMO_cyclopentadiene_–LUMO_cycloalkene_, and the inverse electron demand (IED), LUMO_cyclopentadiene_–HOMO_cycloalkene_, orbital interactions are the most stabilizing for the DA reaction between cyclopentadiene and cyclopropene. The NED orbital energy gap for cyclopropene is the smallest and, hence, more stabilizing than for cyclohexene (Fig. 8a). On top of that, the NED orbital overlap is also larger for cyclopropene than for cyclohexene, due to weaker primary orbital interactions from the increasingly more delocalized π* orbitals over the adjacent atoms in the LUMO_cycloalkene_ as the ring size of the cycloalkene increases. In addition, the IED orbital overlap is also larger for cyclopropene and drops significantly for cyclohexene as a result of the loss in secondary orbital interactions (SOI; Fig. 8b). Cyclopropene has an additional stabilizing orbital overlap contributing to the IED orbital interaction, which involves the pseudo-π-CH_2_ lobes that is able to overlap with the LUMO_cyclopentadiene_ (Fig. 8c). As the ring size of the cycloalkene increases, the pseudo-π-CH_2_ lobes of its HOMO_cycloalkene_ bend increasingly outwards, away from the incoming cyclopentadiene and, therefore, cannot efficiently overlap with the LUMO_cyclopentadiene_, that is, the SOI fades out for the larger cycloalkenes, such as cyclohexene (Fig. 8c). In all, it is the combination of primary and secondary orbital interactions that, ultimately, determine the reactivity trend in the DA reaction between cyclopentadiene and cycloalkene, when the ring size of the cycloalkene increases.

### 4.2. Cycloaddition reactivity of cycloalkynes

**Fig. 9:**
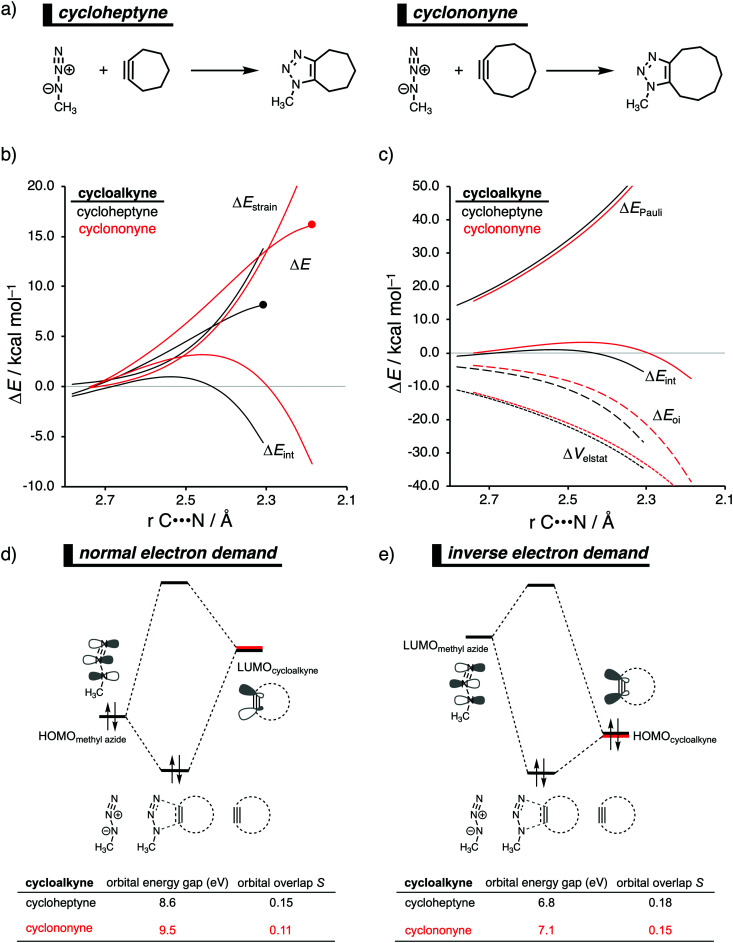
(a) Computationally analyzed 1,3-dipolar cycloaddition reactions between methyl azide and cycloheptyne (left) and cyclononyne (right). (b) Activation strain analysis and (c) energy decomposition analysis of the 1,3-dipolar cycloaddition reaction of methyl azide with cyclohepyne and cyclononyne, where the energies are projected onto the average distance of the two newly forming C⋯N bonds (TS indicated with dot). Molecular orbital diagram with the key orbital energy gap and overlap of the (d) HOMO_methyl azide_–LUMO_cycloalkyne_ normal electron demand (NED) interaction and (e) LUMO_methyl azide_HOMO_cycloalkyne_–LUMO_methyl azide_ inverse electron demand (IED) interaction obtained at consistent geometries with an average C⋯N bond-forming length of 2.22 Å, computed at M06-2X/TZ2P//M06-2X/6-31+G(d).

In analogy with the Diels–Alder reaction involving cycloalkene, the 1,3-dipolar cycloaddition reactivity between methyl azide and cycloalkynes increases as the ring size decreases. The cycloaddition of cycloheptyne is predicted to proceed rapidly with a low reaction barrier (*via* an early transition state) and is highly exergonic. The cycloaddition with the larger cyclononyne, on the other hand, has not only a higher reaction barrier but is also less exergonic. By applying the activation strain model, we were able to pinpoint the physical factors that control these reactivity trends upon changing the cycloalkynes ring size.^14*b*^ Interestingly, we found that the strain energy for both reactions is nearly identical and, therefore, not responsible for their differences in reactivity (Fig. 9b). We want to emphasize that the pre-distorted geometry of cycloalkynes, to which the enhanced reactivity is commonly attributed to,^36^ is only the driving force behind the enhanced cycloaddition reactivity when going from linear to cyclic alkynes and not when reducing the ring size. Instead, the origin of the increase in reactivity as the ring size of the cycloalkynes decreases originates from differences in the enhanced interaction energy. Due to the decisive role of the interaction energy, this term was analyzed in more detail using the EDA method. The EDA diagram in Fig. 9c shows that the reaction involving cycloheptyne goes with the most stabilizing orbital interactions and, therefore, also the most favorable interaction energy. Differences in the Pauli repulsion and electrostatic interaction curves are minimal or even opposite from the trend in interaction energy. Thus, the important finding is that increased pre-distortion of the cycloalkyne, as the ring size decreased, enhances the orbital interaction and does not lead to a decrease in strain energy.

With the help of our detailed Kohn–Sham molecular orbital analysis, we were able to pinpoint the origin of the difference in orbital interactions that emerges from our EDA. We found that the 1,3-dipolar cycloaddition involving cycloheptyne benefits from a more stabilizing normal electron demand (NED), HOMO_methyl azide_–LUMO_cycloalkyne_, as well as a more favorable inverse electron demand (IED), HOMO_methyl azide_–LUMO_cycloalkyne_, orbital interaction. Cycloheptyne engages in the strongest NED interaction because it has the smallest NED orbital energy gap and greatest orbital overlap (Fig. 9d). As the ring size increases to cyclononyne, the NED orbital energy gap also increases, because a less bend alkyne has, due to a reduced repulsive intra-molecular orbital overlap, a more stable LUMO_cycloalkyne_.^37^ In addition, there is a continuous decrease in NED orbital overlap upon increasing the ring size (Fig. 9e). When the cycloalkyne bends more, *i.e.*, small ring size, the LUMO_cycloalkyne_ extends further toward the incoming methyl azide, which results in a more favorable orbital overlap with the HOMO_methyl azide_.^38^ Thus, cycloadditions involving cyclononyne proceed with a diminished orbital overlap, due to smaller alkyne distortion compared to cycloheptyne. Furthermore, the IED orbital interaction follows the same trend, namely, cycloheptyne has the smallest IED orbital energy gap and most favorable. The HOMO of cyclononyne is more stable relative to cycloheptyne. This leads to larger less stabilizing IED orbital energy gap for cyclononyne. The orbital overlap for cyclononyne is also less stabilizing relative to cycloheptyne. In summary, the accelerated reactivity of smaller cycloalkynes is not due to their more advanced pre-distortion towards the transition state geometry, but because of their enhanced orbital interactions that are a direct result of the smaller NED and IED orbital energy gap and better orbital overlap.

### 4.3. Cycloaddition reactivity of cycloallenes

**Fig. 10:**
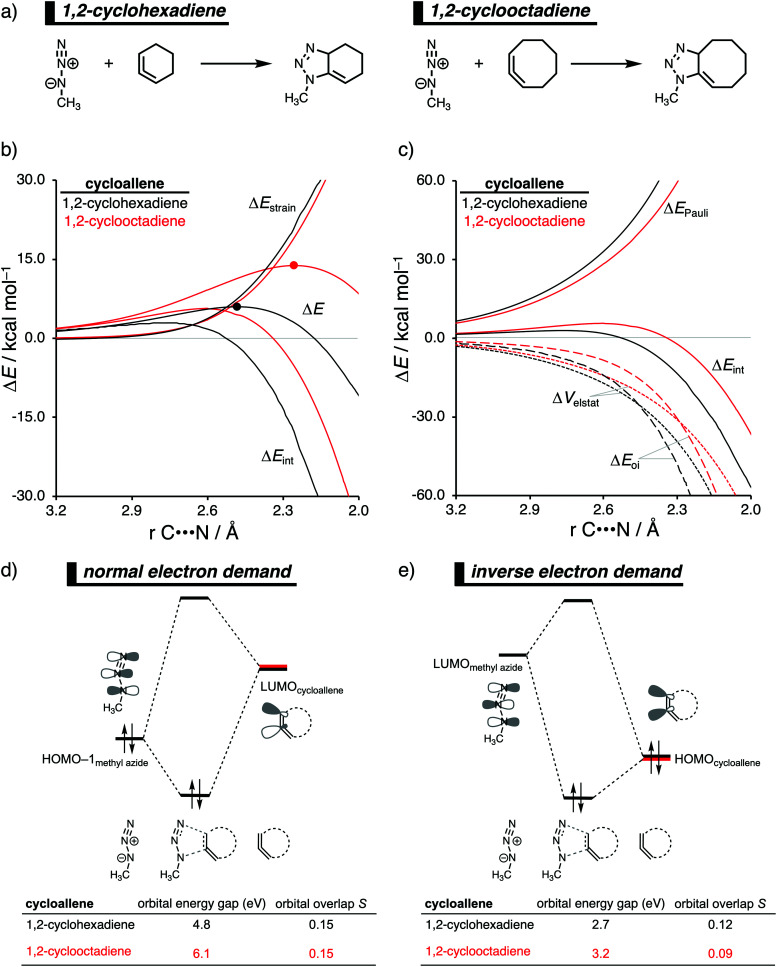
(a) Computationally analyzed 1,3-dipolar cycloaddition reactions between methyl azide and 1,2-cyclohexadiene (left) and 1,2-cyclooctadiene (right). (b) Activation strain analysis and (c) energy decomposition analysis of the 1,3-dipolar cycloaddition reaction of methyl azide with 1,2-cyclohexadiene and 1,2-cyclooctadiene, where the energies are projected onto the average distance of the two newly forming C⋯N bonds (TS indicated with dot). Molecular orbital diagram with the key orbital energy gap and overlap of the (d) HOMO_methyl azide_–LUMO_cycloallene_ normal electron demand (NED) interaction and (e) HOMO_cycloallene_–LUMO_methyl azide_ inverse electron demand (IED) interaction obtained at consistent geometries with an average C⋯N bond-forming length of 2.48 Å, computed at BP86/TZ2P.

The 1,3-dipolar cycloaddition reactivity between methyl azide and cycloallenes follows the same reactivity trend as the corresponding cycloalkyne, namely, the reactivity increases when the ring size decreases. An activation strain analysis was performed to pinpoint the intrinsic differences in reactivity between 1,2-cyclohexadiene and 1,2-cyclooctadiene in the 1,3-dipolar cycloaddition with methyl azide.^14*c*^ Our results clearly show that the origin of the increased reactivity as the ring size of cycloallene decreases can be entirely attributed to the differences in interaction energy, which becomes increasingly more stabilizing from along this series (Fig. 10b). The strain energy, on the other hand, is for all studied cycloallenes nearly the same. As expected upon decreasing the size of the ring, the cycloallene becomes more pre-distorted towards the cycloaddition reaction with Az, which leads to a smaller contribution of the deformation of the smaller cycloallene to the total strain energy, in line with earlier studies.^39^ The contribution of the deformation of methyl azide to the strain energy, however, becomes larger when the ring size decreases, because the more reactive cycloallene deforms, due to a stronger interaction, methyl azide to a larger degree. These two contributors to the strain energy counteract each other, resulting in a nearly identical strain energy upon varying the ring size. The origin of the differences in interaction energy is uncovered by means of the EDA (Fig. 10a). This analysis method reveals that the orbital interactions are the driving force behind the trend in interaction energy, guided by a smaller contribution of the electrostatic interactions. The Pauli repulsion, on the other hand, shows a reverse trend, and is, therefore, not responsible for the trend in interaction energy.

To further probe the key orbital interactions, that cause the difference in orbital interactions as shown in our energy decomposition analysis (Fig. 10b), involved in the 1,3-dipolar cycloadditions of methyl azide with 1,2-cyclohexadiene and 1,2-cyclooctadiene, we analyzed the molecular orbitals participating in these interactions. The molecular orbitals participating in the normal electron demand (NED), HOMO−1_methyl azide_–LUMO_cycloallene_, and inverse electron demand (IED), LUMO_methyl azide_–HOMO_cycloallene_, reveal that the more stabilizing orbital interactions when the ring size of the cycloallene increases is predominantly determined by a reduction in orbital energy gap. The least reactive cycloallene, 1,2-cyclooctadiene, has the largest and least favorable NED orbital energy gap (Fig. 10d). As the ring size decreases from an eight- to a six-membered ring, the NED orbital energy gap continuously decreases, resulting in the smallest and most favorable orbital energy gap for 1,2-cyclohexadiene. The orbital overlap for the NED interaction is, however, identical for both reactions. Furthermore, the IED interaction involving 1,2-cyclooctadiene has the largest and, therefore, least favorable IED orbital energy gap, which decreases and making it more favorable for 1,2-cyclohexadiene (Fig. 10e). The reduction of both the NED and IED orbital energy gaps can directly be related to the pre-distortion of the cycloallene, because a more bent cycloallene, *i.e.*, smaller ring, goes with a smaller HOMO–LUMO gap in the π-electron system and, therefore, engages in stronger donor–acceptor orbital interactions with methyl azide. Thus, the enhanced reactivity of smaller cycloallenes originates from the increased predistortion of these allenes, leading to a systematically lower reaction barrier not due to the expected variations in the destabilizing strain energy, but, instead, from their differences in the stabilizing interaction energy.

## 5. Conclusions

The activation strain model (ASM) in combination with Kohn–Sham molecular orbital (KS-MO) theory is a universal approach for establishing a causal relationship between, on one hand, the electronic structure and rigidity of reactants and, on the other hand, their reactivity. Our unified approach does not simply rely on the generation of numerical data (*easy task*), but actually provides meaning and insight into causal relationships behind numerical data (*challenging, but rewarding, task*). In this Feature Article, we have showcased the general applicability of the activation strain model to explain the reactivity and selectivity of a wide range of chemical reactions including (i) the competition between S_N_2 and E2 pathways, (ii) small molecule activation by main-group metallylenes, and (iii) the cycloadditions of cycloalkenes, cycloalkynes, and cycloallenes.

First, we saw that the Lewis acid–base interaction between the Lewis base (protophile or nucleophile) and the substrate determines the outcome of the S_N_2 *versus* E2 competition. The S_N_2 pathway is preferred with a weak Lewis base (low-lying HOMO) because of the weak interaction with the substrate (high-lying LUMO) is unable to overcome the high characteristic distortivity of the E2 pathway. The E2 pathway, on the other hand, is preferred with a strong Lewis base (high-lying HOMO) because the more stabilizing interaction with the more distorted substrate (low-lying LUMO, *i.e.*, high TS acidity) can overcome the strain associated with breaking two bonds and determines the reactivity trend.

Next, we saw that model metallylenes can efficiently activate H_2_. The barrier for H_2_ activation increases as the group 14 metallylene atom is varied from carbon to tin due to the less stabilizing the orbital interactions attributed to less efficient donation of electrons from the lone pair orbital of the metallylene (HOMO_metalyllene_) into the σ*-orbital of H_2_ (LUMO_H2_). Along the series, from carbon to tin, the HOMO_metalyllene_ becomes more stable (lower-lying HOMO) and more diffuse, *i.e.*, increased spatial extent of the lone pair orbital on the central atom of the metallylene, which leads to a larger HOMO_metallylene_–LUMO_H2_ energy gap and a poorer orbital overlap with H_2_.

Finally, we saw that the enhanced cycloaddition reactivity of small-ring cycloalkenes, cycloalkynes, and cycloallenes compared to their large-ring analogs or linear counterparts, originates from their pre-distorted geometry. The pre-distortion imparted by the ring does not reduce the strain energy required to obtain the transition state geometry, but, instead, enhances the orbital interactions. For the cycloalkene, a smaller ring leads to additional stabilizing secondary orbital interactions. A higher degree of bending in the cycloalkyne and cycloallene, in contrast, results in a higher-lying HOMO, a lower-lying LUMO, and a greater spatial extent of the orbital on the face that reacts.

Thus, these recent examples exhibit the powerful combination of the activation strain model and molecular orbital theory to unravel the underlying factors that control chemical reactivity. These insights are then useful for the rational design and tuning of novel more selective transformations.

## Conflicts of interest

There are no conflicts to declare.

## References

[cit1] (a) Szabo A. and Ostlund N. S., Modern Quantum Chemistry, Dover Publications Inc., New York, 1996

[cit2] Fukui K. (1971). Acc. Chem. Res..

[cit3] Shaik S. and Hiberty P. C., A Chemist's Guide to Valence Bond Theory, Wiley-Interscience, Hoboken, New Jersey, 2007

[cit4] Marcus R. A. (1964). Annu. Rev. Phys. Chem..

[cit5] Sun X., Soini T. M., Poater J., Hamlin T. A., Bickelhaupt F. M. (2019). J. Comput. Chem..

[cit6] Bickelhaupt F. M. (1999). J. Comput. Chem..

[cit7] Vermeeren P., Hamlin T. A., Fernández I., Bickelhaupt F. M. (2020). Angew. Chem., Int. Ed..

[cit8] Wolters L. P., Bickelhaupt F. M. (2015). Chem. – Asian J..

[cit9] Hamlin T. A., Fernández I., Bickelhaupt F. M. (2019). Angew. Chem., Int. Ed..

[cit10] van Zeist W. J., Bickelhaupt F. M. (2010). Org. Biomol. Chem..

[cit11] Vermeeren P., van der Lubbe S. C. C., Fonseca Guerra C., Bickelhaupt F. M., Hamlin T. A. (2020). Nat. Protoc..

[cit12] Vermeeren P., Hansen T., Jansen P., Swart M., Hamlin T. A., Bickelhaupt F. M. (2020). Chem. – Eur. J..

[cit13] Vermeeren P., Doppert M. T., Bickelhaupt F. M., Hamlin T. A. (2021). Chem. Sci..

[cit14] Levandowski B. J., Hamlin T. A., Bickelhaupt F. M., Houk K. N. (2017). J. Org. Chem..

[cit15] Ess D. H., Houk K. (2007). J. Am. Chem. Soc..

[cit16] Fukui K. (1981). Acc. Chem. Res..

[cit17] Stasyuk O. A., Sedlak R., Fonseca Guerra C., Hobza P. (2018). J. Chem. Theory Comput..

[cit18] (a) Hamlin T. A. , Vermeeren P., Fonseca Guerra C. and Bickelhaupt F. M., in Complementary Bonding Analysis, ed. S. Grabowsky, De Gruyter, Berlin, 2021, pp. 199–212

[cit19] (c) Baerends E. J. , et al., ADF, SCM, Theoretical Chemistry, Vrije Universiteit, Amsterdam, The Netherlands, 2013, http://www.scm.com

[cit20] (b) Albright T. A. , Burdett J. K. and Wangbo W. H., Orbital Interactions in Chemistry, Wiley, New York, 2013

[cit21] Fonseca Guerra C., Handgraaf J. W., Baerends E. J., Bickelhaupt F. M. (2004). J. Comput. Chem..

[cit22] Ziegler T., Rauk A. (1979). Inorg. Chem..

[cit23] Caldeweyher E., Bannwarth C., Grimme S. (2017). J. Chem. Phys..

[cit24] Hamlin T. A., Swart M., Bickelhaupt F. M. (2018). ChemPhysChem.

[cit25] Gronert S. (2001). Chem. Rev..

[cit26] van Zeist W.-J., Ren Y., Bickelhaupt F. M. (2010). Sci. China: Chem..

[cit27] Acree Jr. W. E. and Chickos J. S., in NIST Chemistry WebBook, NIST Standard Reference database Number 69, ed. P. J. Linstrom and W. G. Mallard, National Institute of Standards and Technology, Gaithersburg MD

[cit28] Wladkowski B. D., Brauman J. I. (1992). J. Am. Chem. Soc..

[cit29] Gronert S. (1991). J. Am. Chem. Soc..

[cit30] (c) Crotti P. and Pineschi M., in Aziridines and Epoxides in Organic Synthesis, Wiley, Hoboken, 2006, pp. 271–313

[cit31] Long F. A., Pritchard J. G. (1956). J. Am. Chem. Soc..

[cit32] Power P. P. (2010). Nature.

[cit33] Bourissou D., Guerret O., Gabbai F. P., Bertrand G. (2000). Chem. Rev..

[cit34] Frey D. G., Lavallo V., Donnadieu B., Schoeller W. W., Bertrand G. (2007). Science.

[cit35] Agard N. J., Prescher J. A., Bertozzi C. R. (2004). J. Am. Chem. Soc..

[cit36] Liu F., Liang Y., Houk K. N. (2017). Acc. Chem. Res..

[cit37] Strozier R. W., Caramella P., Houk K. N. (1979). J. Am. Chem. Soc..

[cit38] Inagaki S., Fujimoto H., Fukui K. (1976). J. Am. Chem. Soc..

[cit39] Barber J. S., Styduhar E. D., Pham H. V., McMahon T. C., Houk K. N., Garg N. K. (2016). J. Am. Chem. Soc..

